# Decoding force production of skeletal muscle from the female brain using functional near-infrared spectroscopy

**DOI:** 10.1186/s13104-023-06588-5

**Published:** 2023-11-01

**Authors:** Hojeong Kim

**Affiliations:** 1 Division of Biotechnology, Institute of Convergence Research, DGIST, Daegu, Republic of Korea; 2 Department of Interdisciplinary Studies, DGIST, Daegu, Republic of Korea

**Keywords:** Neural decoding, Cortical activation, Muscle force, Female brain, fNIRS

## Abstract

**Objective:**

Noninvasive neural decoding enables predicting motor output from neural activities without physically damaging the human body. A recent study demonstrated the applicability of functional near-infrared spectroscopy (fNIRS) to decode muscle force production from hemodynamic signals measured in the male brain. However, given the sex differences in cerebral blood flow and muscle physiology, whether the fNIRS approach can also be applied to the female brain remains elusive. Therefore, this study aimed to evaluate whether fNIRS can be used to identify the optimal cortical region and hemodynamic predictor to decode muscle force output in females.

**Results:**

Statistical group analysis for eight healthy female adults showed that the cortical region for wrist control was topologically dorsal to that for finger control over the primary sensorimotor cortex. This cortical area was maximally activated while the wrist flexor muscles were contracted to hold a load on the subject’s palm, as was the case for males. However, the dynamics of oxyhemoglobin concentration measured from the most activated cortical area differed between females and males. The signal intensity during 100% maximal voluntary contraction and the signal increase rate at 50% maximal voluntary contraction was lower and faster in females. Eight predictors were used to characterize hemodynamic signals’ amplitude and temporal variation in the female cortex. Unlike the case for males, only the trajectory predictors for the amplitude of oxyhemoglobin concentration change were strongly correlated with the strengths of force produced by the wrist flexor muscles, showing a linear relationship. These results suggest gender-specific hemodynamics must be considered for decoding low-level motor control with fNIRS in females.

## Introduction

In neural decoding, the information encoded in the electrical activity of individual cells or networks of neurons is extracted. The accuracy of neural decoding is crucial in assessing rehabilitation outcomes and neurological diagnostics via neural interfaces. For motor systems, both the spatial and temporal patterns of cortical activities are necessary to accurately decode cortical activation to identify the desired motor output in humans [1].

Motor outputs have been decoded by noninvasively measuring cortical activation from the human brain under two modalities. Electrical signals related to neuronal activities have been characterized through electroencephalography (EEG) [2]. This approach has been focused mainly on dynamical characteristics of cortical activation over a broad brain area due to its excellent temporal resolution (millisecond range) but poor spatial resolution (approximately 6–9 cm) [3]. Hemodynamic signals related to neurovascular coupling have been measured with functional magnetic resonance imaging (fMRI) [4, 5]. This method has been focused mainly on structural characteristics of cortical activation during simple motor tasks due to its poor temporal resolution (> a few seconds) and significant movement artifacts [6, 7]. Alternatively, the functional near-infrared spectroscopy (fNIRS) has been used for spatiotemporal analysis of cortical activation for a wide range of cognitive [8, 9] or movement tasks [10, 11] due to its relatively high resolution for time (up to 100 ms) and space (1–3 cm) along with its high resistance to movement artifacts, low cost, and low noise level.

Recent studies have further demonstrated that the fNIRS may be applicable to decode low-level motor control, such as submaximal [12] or maximal muscle contraction, based on hemodynamic signals measured over the motor cortex for the male brain [13]. However, sex differences have been reported in the physiological studies of cerebral blood flow [14] and skeletal muscle [15]. In addition, studies using EEG and fMRI have shown significant differences in spatiotemporal cortical activation patterns between males and females during hand movements [16, 17]. Thus, whether the fNIRS approach can also be applied to decode cortical activation for low-level motor tasks in females remains to be studied.

Here, we evaluated whether the fNIRS technique can be used to decode the force production of skeletal muscle from oxyhemoglobin dynamics in the female brain. The spatial and temporal dynamics of oxyhemoglobin signals were measured and analyzed to extract the optimal cortical region and coding scheme for force control of wrist flexor muscle in the female brain. The results demonstrated the feasibility of decoding the force production by skeletal muscle in females but in a different way from males. This study may contribute to the establishment of sex-specific neural interfaces for neurorehabilitation and neurological diagnostics.

## Methods

DGIST Ethics Committee approved this human study (DGIST_180202_HR_-001-01). We conducted human experiments according to the Declaration of Helsinki. Eight right-handed healthy female adults (age 21 ± 1.7 years; weight 53.5 ± 5.4 kg; height 160.5 ± 3.3 cm) were recruited who did not have any neurological, physical, or psychiatric disease history. The sample size was estimated using the IBM SPSS Statistics version 27 (IBM Corp., Armonk, N.Y., USA) with the following conditions: one-sample t-test, power of 0.8, population mean of 5, population standard deviation of 5, and one-side analysis with significance level of 0.05. The subject group was relatively homogeneous and selected with no bias. We gave all subjects written informed consent before participating in the experiment. The experimental procedures and data analysis used for this study have been fully addressed in our previous study for males [13].

Briefly, the subjects lay on a customized bed that allowed their body to be fully supported except for their wrist and hand, isolating the wrist flexor muscles. A block design was applied to each trial, comprising three stages of rest (30 s), task (30 s), and rest (30 s). The subject conducted the same trial three times in a row over one session. A session was begun after 20 s rest to stabilize the hemodynamic signals. During the stage of motor task, the subject holds a specific load on her right palm while keeping the wrist straight, ensuring the isometric condition. The motor task was started and ended by having their right forearm touched. We determined the wrist flexor muscles’ maximal voluntary contraction (MVC) by increasing the load until the subject could not maintain it. Three sessions were carried out for each subject at 0%, 50%, and 100% MVC. At least 5 min was taken for muscle relaxation between sessions to avoid influences of fatigue and injury. During the experiment, the subjects were blindfolded and earplugged to minimize reactions evoked by visual and auditory stimuli.

**Fig. 1:**
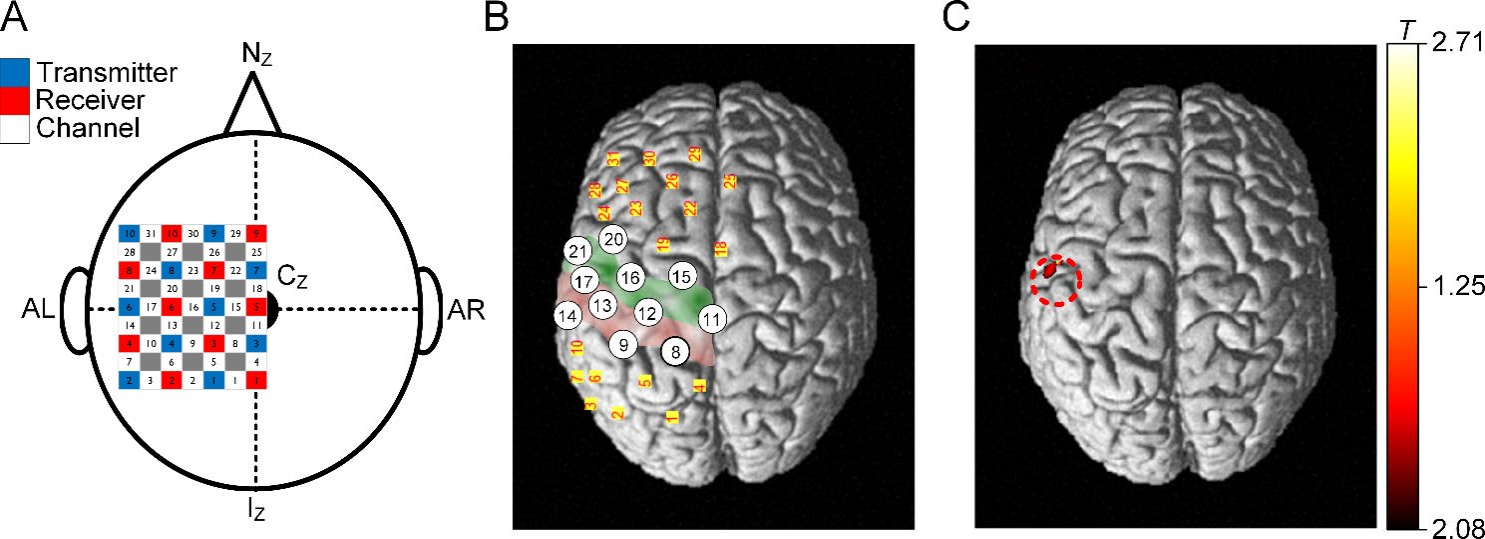
Identification of the most activated area on the primary sensorimotor cortex during maximal contraction of the wrist flexor muscles under isometric conditions. **(A)** Arrangement of optodes and channels over the head. Nz, Cz, AL, AR, and Iz indicate the nasion, central point, left preauricular point, right preauricular point, and inion, respectively. **(B)** Anatomical locations of channels in the left hemisphere. The green and red areas indicate the primary motor and somatosensory regions. Circled numbers indicate channels correlated with the primary somatosensory and motor areas. **(C)** Statistical group analysis for cortical activation across all subjects. A color code indicating a higher T value with a brighter color was applied to represent the relative cortical activation during the motor task. The most activated cortical area was identified during the motor task at 100% MVC (uncorrected p-value < 0.05). The dashed circle indicates the cortical area maximally activated during the motor task in males. This data was adopted from the previous study (Fig. 4 in [13])

We used a commercially available fNIRS system (FOIRE-3000, Shimadzu Co., Kyoto, Japan) with the default settings for wavelength (780, 805, and 830 nm) and sampling (30.303 Hz) over the left-brain hemisphere. The oxyhemoglobin concentration change (∆Hb_oxy_) was estimated by the modified Beer-Lambert law with the default coefficient values set in the fNIRS system. The left hemisphere’s primary somatosensory and motor cortex were covered by a 5-by-4 array of twenty optodes (ten transmitters and ten receivers) (see Fig. 1A for graphical illustration). Individual optodes were placed in respective holders apart by 3 cm on a commercially available elastic cap, including a chin strap. The 3-dimensional coordinates of individual optodes were measured after the experiments using a 3-dimensional digitizing system (FASTRAK, Polhemus, VT, USA) and projected over the brain image rendered in the 3-dimensional space using the NIRS-SPM software (version NIRS-SPM_V4_r1 and spm8) [18]. The Brodmann areas (MRIcro) optically measured over the cerebral cortex were statistically estimated using the NIRS-SPM software. All channels related to the primary somatosensory and motor cortex were analyzed.

Raw hemodynamic signal data were measured three times in a session and averaged using the LABNIRS system. The averaged data were preprocessed, including detrending with a discrete cosine transformation and bandpass filtering with a cutoff frequency of 1/128 Hz for high-pass filtering and a hemodynamic response function for low-pass filtering using the NIRS-SPM software. A linear baseline correction was made to remove longitudinal signal drift. Consequently, individual time series of filtered data were shifted such that their values at task onset were set to zero. We further normalized the corrected signal for unbiased comparison between subjects and channels. The normalization was conducted by dividing the corrected signal by the standard deviation calculated for 10 s before the initiation of motor task.

**Fig. 2:**
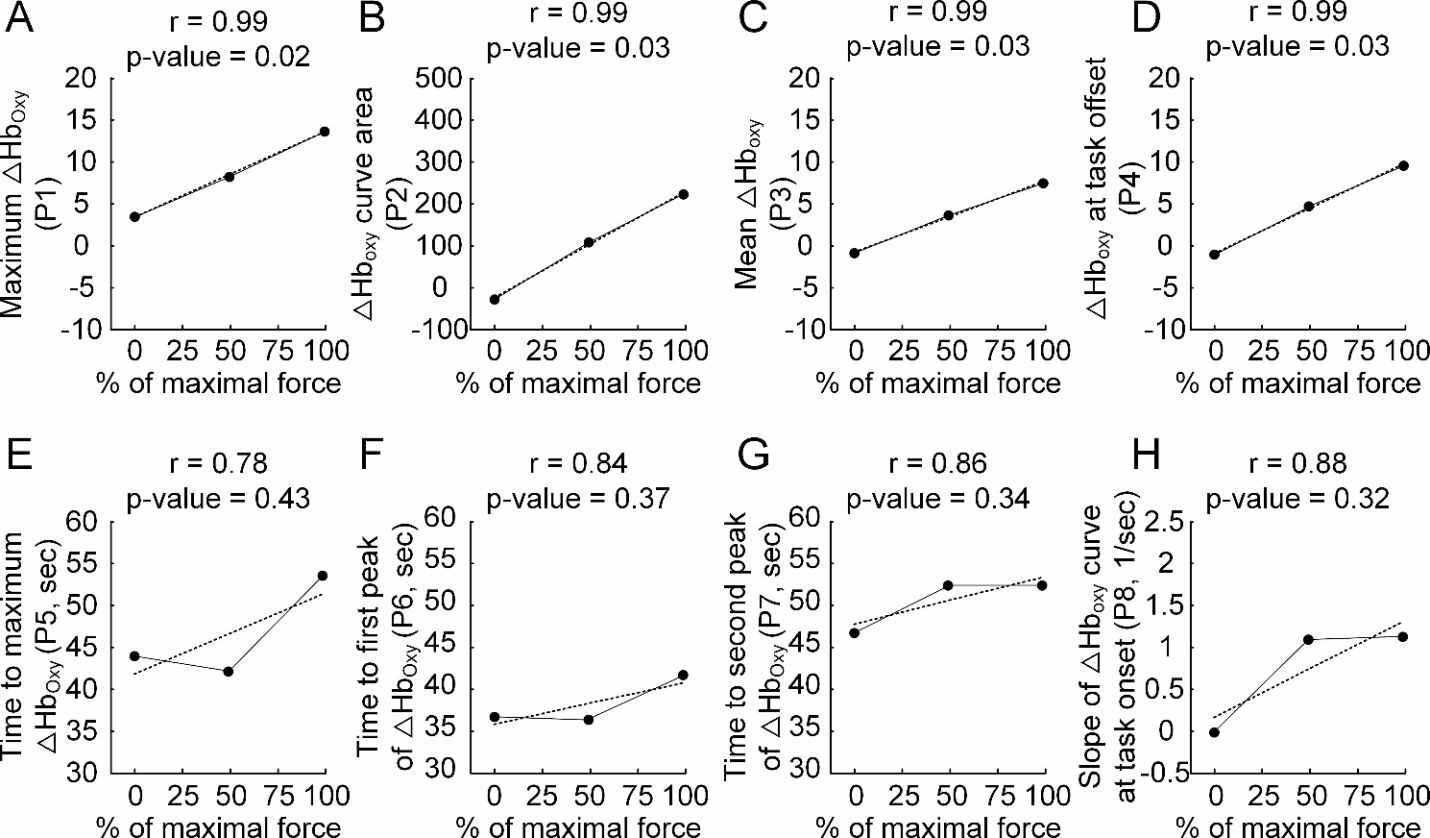
Correlation between the trajectory predictor and muscle force production. **A-H.** Each trajectory predictor for the ∆Hb_oxy_ signal from channel 21 was plotted against the force production varying from 0 to 100% MVC under isometric conditions. The trajectory predictor values measured from individual subjects were averaged for all subjects. The mean values of individual predictors were indicated with solid dots (black) and fitted to the linear regression line indicated by dotted lines (black). The regression equation and r^2^ value for each predictor are 0.1*x + 3.34 and 0.99 for P1, 2.52*x-23.53 and 0.99 for P2, 0.08*x-0.78 and 0.99 for P3, 0.11*x-0.92 and 0.99 for P4, 0.1*x + 41.76 and 0.61 for P5, 0.05*x + 35.77 and 0.7 for P6, 0.06*x + 47.69 and 0.75 for P7, and 0.01*x + 0.16 and 0.77 for P8, respectively, where x indicates the percentage of maximal muscle force

The cortical areas activated during the motor task were statistically identified using the functions (i.e., general linear model and continuous random field) built in the NIRS-SPM software. The changes in hemodynamic signal from the maximally activated cortical area were analyzed to predict the level of voluntary muscle contraction. As reported in the previous study [13], the hemodynamic signal trajectory was characterized by eight predictors. Four predictors represented the magnitude of oxygenated hemoglobin concentration (i.e., P1-P4 in Fig. 2A and D, respectively). Others described the variation in oxygenated hemoglobin concentration over time (i.e., P5-P8 in Fig. 2E H, respectively). The predictor values obtained for each subject were averaged across all subjects for 0%, 50%, and 100% MVC, respectively.

We assessed how the predictor’s grand mean represented the voluntary contraction force by calculating the Pearson coefficient (r) and p-value (two-tailed test) using the IBM SPSS Statistics version 27. The trajectory predictor-voluntary contraction relationship was fitted with a linear regression line, and the r^2^ value was used to compare the goodness of fit under the SPSS software environment. The statistical significance was determined by p-value < 0.05. The data from our previous study for males [13] were adapted for the purpose of comparison in this study.

## Results

We first identified the cortical area responsible for controlling isometric contraction of the right wrist flexor muscles. The group analysis of cortical activation was statistically performed for the primary sensorimotor cortex of the left hemisphere during the motor task using the NIRS-SPM software. Figure 1 shows the cortical area and corresponding channel location that were maximally activated for the motor task at 100% MVC. Peak cortical activation was observed around channel 21, indicating the cortical area in the left hemisphere that controlled the force produced by the wrist flexor muscles in isometric conditions. This cortical area was overlapped with that for males (see a dashed circle in Fig. 1C). Channel 21 was chosen for further analysis of the correlation between cortical activation and force generation for the wrist flexor muscles.

**Fig. 3:**
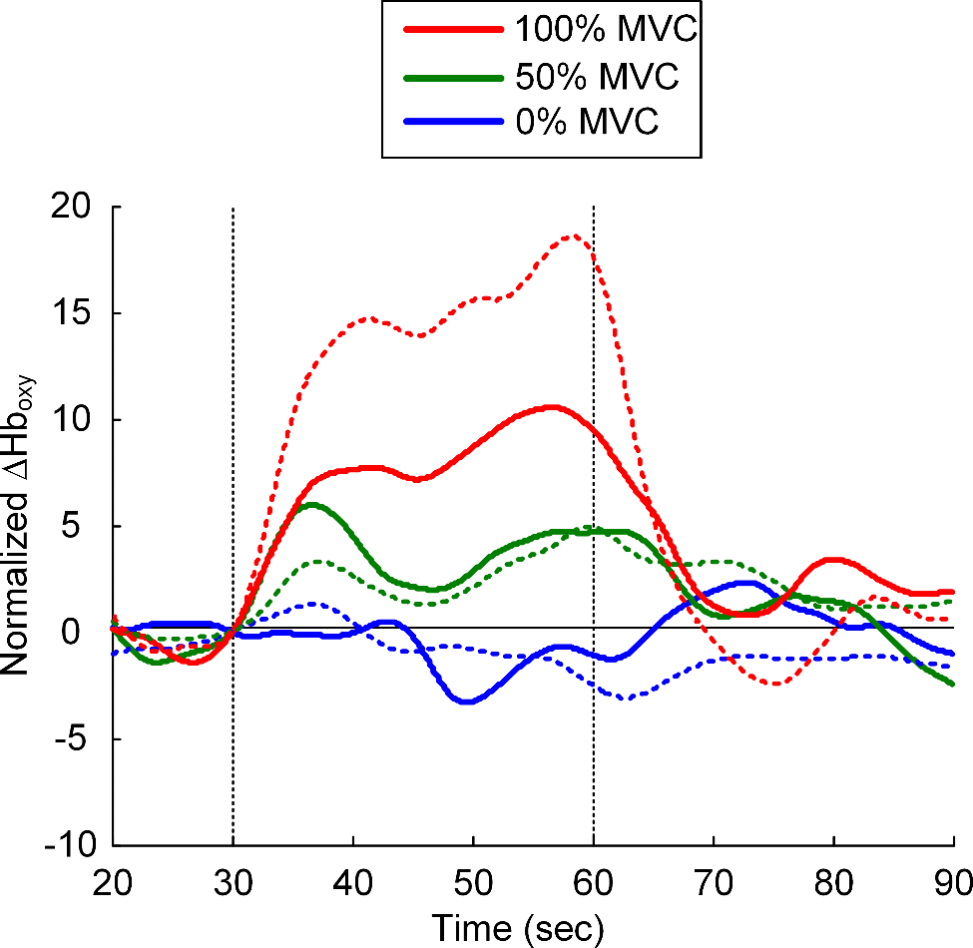
Time course of the grand average ∆Hb_oxy_ signal measured from channel 21. The normalized ∆Hb_oxy_ data collected from channel 21 during the motor task were averaged across all subjects and represented by a solid line. The color of the solid line indicates the level of voluntary muscle contraction (0, 50, and 100% MVC). For the purpose of comparison, the previously published data for males (Fig. 5A in [13]) were superimposed as dashed lines. The start and end of voluntary muscle contraction were indicated by the vertical dotted lines (black). The baseline corrected for the ∆Hb_oxy_ signal was indicated by the horizontal solid line (black)

We then analyzed how the trajectory of ∆Hb_oxy_ signal changed with the voluntary contraction level. Figure 3 shows the statistical summary (i.e., mean) for the ∆Hb_oxy_ signal data obtained from the cortical region nearby channel 21 at three levels of voluntary contraction (0%, 50%, and 100% MVC). The intensity and the increase rate for the ∆Hb_oxy_ signal were increased as the strength of voluntary force enlarged from 0 to 100% MVC. These results suggest considering both amplitude and temporal variation of the hemodynamic signal for decoding the force control of wrist muscles by the female corticospinal neurons. However, the increase rate of ∆Hb_oxy_ signal tended to be faster in females than in males for the task at 50% MVC (green lines in Fig. 3). The intensity of ∆Hb_oxy_ signal tended to be lower in females than males for the task at 100% MVC (red lines in Fig. 3).

Lastly, we investigated the relationship between each trajectory predictor for hemodynamic signals and voluntary contraction level for wrist muscles. Eight trajectory predictors were considered for the maximum ∆Hb_oxy_ value (P1), area under ∆Hb_oxy_ curve (P2), average of ∆Hb_oxy_ values (P3), ∆Hb_oxy_ value at task offset (P4), timing of ∆Hb_oxy_ maximization (P5), timing of first ∆Hb_oxy_ peak (P6), timing of second ∆Hb_oxy_ peak (P7), and slope of ∆Hb_oxy_ curve at task onset (P8). Figure 2 demonstrates how individual predictors correlated with the voluntary muscle force regarding the ∆Hb_oxy_ data measured from the cortical region (nearby channel 21) maximally activated during the motor task at 100% MVC. Overall, the voluntary force level was more strongly correlated with the trajectory predictors for the amplitude variation (i.e., P1-P4) than those for the temporal variation (i.e., P5-P8) in the ∆Hb_oxy_ signal. Any trajectory predictors (i.e., P5-P8) for the temporal variation were not significantly correlated with the level of muscle contraction. The most effective predictor for voluntary muscle contraction was found to be P1 (p-value < 0.05) representing the peak of ∆Hb_oxy_ signal during the task in females.

## Discussion

Using the fNIRS approach, we identified the maximally activated cortical area for contractions of the wrist flexor muscles and its coding scheme for voluntary muscle contraction over a full range in females.

The wrist region dorsal to the finger region on the primary sensorimotor cortex was the most activated while isometrically contracting the wrist flexor muscles in females (red area in Fig. 1C). This result was consistent with those obtained in males using fNIRS (red dashed circle in Fig. 1C). The similarity of the primary sensorimotor cortex area responsible for voluntary force control of wrist muscles between males and females may support the hypothesis that muscles are represented within the motor cortex [19].

Similar to the case for males (see Fig. 5A in [13]), the trajectory of the ∆Hb_oxy_ signal showed a complex form including several peaks during motor tasks (Fig. 3), implying the existence of both amplitude (i.e., firing frequency modulation) and temporal (i.e., firing timing modulation) coding schemes in the female brain [20]. However, a tendency for a faster cortical activation rate at 50% MVC and lower cortical activation intensity at 100% MVC was found in females. This gender-specific temporal and amplitude coding scheme might imply the functional and structural differences in cortical synapses between females and males [21]. In addition, the less cortical activation in females during wrist muscle contraction was contrary to those obtained from EEG studies during hand movement [16] and fMRI studies during finger tapping [17]. The discrepancy between the present and previous studies might indicate differential neural strategies for low- and high-level motor control between females and males.

For both females and males, the amplitude coding scheme was predominant over the temporal coding scheme for wrist muscle contractions. P1 was the most strongly related to the level of voluntary muscle contraction in females (Fig. 2A), whereas P4 was the most effective predictor in males (see Fig. 6D in [13]). However, the temporal coding scheme appeared to differ between females and males. No trajectory predictor (i.e., P5-P8) for the temporal variation of the hemodynamic signal was strongly correlated to the voluntary muscle contraction for females (Fig. 2H). In contrast, P5 was significantly correlated with the voluntary muscle contraction for males (see Fig. 6H in [13]). This result implies the sex discrepancy in the temporal coding scheme that corticospinal neurons used for voluntary muscle contraction between males and females [22].

Unlike males (Fig. 6 in [13]), there was a tendency towards a linear relationship between the hemodynamic signal amplitude (i.e., P1-P4) and muscle force intensity for females (see Fig. 6 in [13] for males). One hypothetical explanation for this result could be that the firing frequency of corticospinal neurons tends to be modulated linearly to produce muscle force in females [23]. However, further investigation would be guaranteed to test the linear relationship between neural responses and oxyhemoglobin concentration regulated by neurovascular coupling during muscle force production in females [24].

In conclusion, fNIRS may be applied to decode muscle force production from cortical hemodynamic activities obtained from the primary sensorimotor cortex in females. The optimal cortical area for decoding muscle force production over the primary sensorimotor cortex appears to be similar between males and females. In females, the force production of skeletal muscle may be the most effectively predicted linearly by the trajectory predictor representing the maximum ∆Hb_oxy_ amplitude during motor activity.

### Limitations

The minimum sampling approach used for this study may limit the statistical power of the results of the present study. Touching the skin to announce the onset of contracting wrist flexor muscles may affect the change in oxygenated hemoglobin concentration at the beginning of force production. The cortical area of investigation was limited to the left hemisphere’s primary somatosensory and motor cortex. Further analysis of changes in deoxygenated hemoglobin concentration would be needed to prevent false positive channel activation.

## Data Availability

The datasets generated during the current study are available from the corresponding author on reasonable request.

## Abbreviations

fNIRS: functional near-infrared spectroscopy
EEG: electroencephalography
fMRI: functional magnetic resonance imaging
∆Hb_oxy_: changes in oxygenated hemoglobin concentration
MVC: maximal voluntary contraction
